# Formation of Covalently Bound Protein Adducts from the Cytotoxicant Naphthalene in Nasal Epithelium: Species Comparisons

**DOI:** 10.1289/ehp.0901333

**Published:** 2009-12-18

**Authors:** Christina DeStefano-Shields, Dexter Morin, Alan Buckpitt

**Affiliations:** Department of Molecular Biosciences, School of Veterinary Medicine, University of California–Davis, Davis, California, USA

**Keywords:** monkey, naphthalene, nasal epithelium, protein adducts, rat, reactive metabolites, species comparisons

## Abstract

**Background:**

Naphthalene is a volatile hydrocarbon that causes dose-, species-, and cell type–dependent cytotoxicity after acute exposure and hyperplasia/neoplasia after lifetime exposures in rodents. Toxicity depends on metabolic activation, and reactive metabolite binding correlates with tissue and site susceptibility.

**Objectives:**

We compared proteins adducted in nasal epithelium from rats and rhesus macaques *in vitro*.

**Methods:**

Adducted proteins recovered from incubations of nasal epithelium and ^14^C-naphthalene were separated by two-dimensional (2D) gel electrophoresis and imaged to register radioactive proteins. We identified proteins visualized by silver staining on complementary nonradioactive gels by peptide mass mapping.

**Results:**

The levels of reactive metabolite binding in incubations of rhesus ethmoturbinates and maxilloturbinates are similar to those in incubations of target tissues, including rat septal/olfactory regions and murine dissected airway incubations. We identified 40 adducted spots from 2D gel separations of rat olfactory epithelial proteins; 22 of these were nonredundant. In monkeys, we identified 19 spots by mass spectrometry, yielding three nonredundant identifications. Structural proteins (actin/tubulin) were prominent targets in both species.

**Conclusions:**

In this study we identified potential target proteins that may serve as markers closely associated with toxicity. The large differences in previously reported rates of naphthalene metabolism to water-soluble metabolites in dissected airways from mice and monkeys are not reflected in similar differences in covalent adduct formation in the nose. This raises concerns that downstream metabolic/biochemical events are very similar between the rat, a known target for naphthalene toxicity and tumorigenicity, and the rhesus macaque, a species similar to the human.

Lung disease is the fourth leading cause of morbidity and mortality in the U.S. population. Although death rates from heart disease, cancer, and cerebrovascular disease have decreased in the past 10 years, death rates from chronic lower respiratory disease have not. Although tobacco use and traffic-related pollutants account for a portion of disease statistics, the precise etiology of lung disease remains unknown. A significant problem in assessing the contribution of individual chemicals or mixtures of chemicals to lung disease is related to the uncertainty of the use of animal models for human lung disease. Numerous chemicals, including naphthalene (Plopper et al. 1992), trichloroethylene (Odum et al. 1992), and styrene (Green et al. 2001), cause differential toxicity in two common rodent models, mice and rats, raising the question of relevance of these rodent models for human risk assessment.

Naphthalene administration results in highly selective necrosis of nonciliated bronchiolar epithelial cells of mice regardless of the route of administration. Inhaled concentrations of 2–5 ppm for 4 hr, well below the current occupational standard, cause detectable toxicity (West et al. 2001). In contrast, injury to airway epithelium of the rat does not occur at concentrations ≤ 100 ppm (West et al. 2001). The nasal epithelium of both rats and mice is highly sensitive to naphthalene administered either parenterally or by inhalation (Lee et al. 2005; Plopper et al. 1992). Recent studies have demonstrated sensitivity of rat olfactory epithelium at concentrations similar to those in urban atmospheres (0.3 ppm) (Dodd et al. 2010).

The species-, tissue-, and site-selective injury caused by naphthalene has been used to identify mechanisms associated with toxicity (for review, see Buckpitt et al. 2002). Naphthalene undergoes metabolism by the cytochrome P450 monooxygenases to reactive metabolites that become bound covalently to tissue proteins. The primary P450 involved in metabolism in the mouse lung and rat nose appears to be cytochrome P450 2F (CYP2F); this protein has high affinity for naphthalene as a substrate (*K*_m,_ ~ 4 μM) with high catalytic turnover (104/min) (Shultz et al. 1999). CYP2F orthologs found in the lungs of nonhuman primates (CYP2F5; Baldwin et al. 2005) and humans (CYP2F1; Nhamburo et al. 1990) had little or no catalytic activity when expressed as recombinant enzyme (Lanza et al. 1999). This was consistent with the finding that lung microsomes from monkeys and humans metabolized naphthalene to water-soluble metabolites at very low rates (Buckpitt and Bahnson 1986; Buckpitt et al. 1992) and that dissected monkey airways generated metabolites very slowly (Boland et al. 2004). Because the initial step in the turnover of naphthalene is critical to toxicity, these data would indicate that monkeys and humans would be considerably less susceptible to naphthalene exposure than rodents. However, more recent data have shown that, although the rates of water-soluble metabolite formation are low, reactive metabolite protein binding occurs at similar levels in sensitive rodent tissues (Cho et al. 1994) and in dissected airways of monkeys (Boland et al. 2004).

Human exposure to naphthalene is nearly universal; the recent National Health and Nutrition Examination Survey studies demonstrated detectable levels of 1-naphthol glucuronide in the urine of all 2,785 subjects tested (Li et al. 2008). However, epidemiologic studies have failed to establish a relationship between naphthalene exposure and any long-term health consequences in the respiratory tract. Either humans are not as susceptible to the compound as rodents are, or respiratory diseases are multifactorial in origin and epidemiologic studies have not been sufficiently powered to detect subtle contributions of naphthalene to the overall incidence of disease.

In the present study we focused on protein-bound metabolites in nasal epithelium of rats and rhesus macaques. In contrast to previous work showing 10- to 100-fold higher rates of naphthalene metabolism by dissected mouse airways compared with the rhesus airway (Boland et al. 2004; Plopper et al. 1991), the present study shows that the rates of formation of covalently bound metabolites in rhesus nasal epithelium are similar to those in rat nasal epithelium, a target for naphthalene toxicity. This study also demonstrates some concordance in the adducted proteins identified in rat and monkey nasal epithelial incubations. We identified proteins involved in the unfolded protein response in incubations with rat but not monkey nasal epithelium. Proteins identified in the present work could serve as potential biomarker targets for studies in exposed human populations.

## Materials and Methods

### Animals

All animal work was conducted under protocols approved by the University of California–Davis (UCD) Animal Use and Care Committee. Animals were treated humanely and with regard to alleviation of suffering. Male Sprague-Dawley rats (200–225 g body weight) were obtained from Harlan Laboratories (Indianapolis, IN) and housed in the UCD vivarium for at least 1 week before experimentation. Animals were euthanized with an overdose of pentobarbital, and olfactory and septal turbinates were removed. Ethmoturbinates and maxilloturbinates were obtained from eight male and seven female rhesus macaques through the California National Primate Research Center [animal characteristics are available in Supplemental Material, Table 1 (doi:10.1289/ehp.0901333)]. All rhesus macaque tissue was from animals euthanized for normal colony maintenance at the center.

### Reagents

We obtained [1,4,5,8-^14^C]naphthalene (specific activity 58 mCi/mmol) from Moravek Radiochemicals (Brea, CA) and checked purity (99%) by high-performance liquid chromatography. Protease inhibitor cocktail III, nondetergent sulfobetaine-195 (NDSB-195), benzamidine, 1,10-phenanthroline, aprotinin, pepstatin, and leupeptin were purchased from Calbiochem (La Jolla, CA). Rhinohide acrylamide/bisacrylamide was from Invitrogen (Carlsbad, CA), and Bio-Rad Protein Assay solution was purchased from Bio-Rad (Hercules, CA). We obtained immobilized pH gradient (IPG) buffers and narrow-range IPG Immobiline dry strips (18 cm) from GE Health Care (Piscataway, NJ). Unless otherwise stated, all reagents were molecular biology grade or better.

### Tissue incubation and preparation

Tissue was incubated in oxygenated Waymouth’s medium deficient in sulfur amino acids (glutathione, cysteine, methionine) containing 250 μM naphthalene or 250 μM ^14^C-naphthalene at a specific activity of 1.10 × 10^5^ disintegrations per minute per nanomole. Incubations were performed for 2 hr at 37°C in a shaking water bath. For protein identification, we incubated tissue with unlabeled substrate.

After the incubation, tissues were rinsed with cold 5% dextrose and homogenized in lysis solution [2 M thiourea, 7 M urea, 4% wt/wt 3-(3-cholamidopropyl)-dimethylammoniopropane sulfonate (CHAPS), 0.5% Triton X-100, 1% dithiothreitol, and 2% protease inhibitor cocktail] using glass–glass homogenizers. Tissues were allowed to sit at room temperature for 1 hr after homogenization to allow further solubilization and denaturation of proteins. Insoluble proteins were removed by centrifugation (100,000 × *g* for 1 hr).

The tough, bony structure of primate turbinates dictated the use of modified procedures for protein recovery. Briefly, after the incubation, the incubation medium was transferred to a clean centrifuge tube. The tissue was washed with 5% dextrose to remove buffer salts, and fractions of the aqueous phase were combined. These samples were centrifuged to recover any tissue/cells removed, and the pellets were combined with the remaining cartilage containing the epithelium. Lysis buffer containing protease inhibitors was added, and the tube was agitated on a rotating shaker to dislodge cells.

### Comparison of covalent binding levels

A portion (~ 1 mg protein) of each sample (from rat or monkey nasal epithelium) incubated with ^14^C-naphthalene was dialyzed in 0.1% sodium dodecyl sulfate, 1 mM disodium EDTA at 4°C [3,500 molecular weight (MW) cutoff Slide-A-Lyzers; Pierce, Rockford, IL]. The dialysis solution was replaced with fresh solution until the counts per minute was less than twice the background level. Finally, we determined the specific activity of adducted protein for each sample by scintillation counting and a protein assay (Bradford 1976).

### Separation and identification of adducted protein

Procedures for the separation, localization, and identification of adducted proteins have been described in detail previously (Lin et al. 2005). Briefly, samples were not pooled, and separate gels were run for each incubation with labeled (for imaging) and unlabeled (for identification) naphthalene. Proteins were resolved by two-dimensional (2D) gel electrophoresis on 18-cm narrow-range pI (isoelectric point) strips for 4.5–5.5 and 5.5–6.7 pI. Strips were rehydrated with 300 μg protein for rat samples or 600 μg protein for monkey samples. Gels containing nonradioactive samples were silver stained and imaged on a 16-bit gray-scale scanner. ^14^C-Labeled sample gels were electroblotted to Sequi-Blot polyvinylidene fluoride membrane (0.2 μm; Bio-Rad), and these were placed against storage phosphor screens for 60 days. The patterns of ^14^C-labeled adducted proteins on the 2D gels were visualized using the GE Typhoon imager (GE Health Care). After excising protein spots of interest from nonradioactive gels, the gel plugs were destained, washed, and incubated in 1:1 (vol/vol) acetonitrile:50 mM ammonium bicarbonate followed by 100% acetonitrile. Tryptic digestion and matrix-assisted laser desorption ionization (MALDI) tandem mass spectroscopy (MS/MS) identification was carried out as described by Lin et al. (2006).

We used GPS Explorer Workstation software (version 3.5; Applied Biosystems, Foster City, CA) and MASCOT (Matrix Sciences, Boston, MA) to search protein databases in the International Protein Index (IPI) rat database (http://www.ebi.ac.uk/IPI/), the National Center for Biotechnology Information primate database (NCBI; http://www.ncbi.nlm.nih.gov/), and the UniProt database (http://www.ebi.ac.uk/uniprot/). Databases were searched for a precursor tolerance of 150 ppm, MS/MS fragment tolerance of 0.2 Da, a maximum of two missed cleavages, and variable modifications for carbamidomethylation and methionine oxidation. An identification with a probability-based MOWSE score > 60, obtained through MASCOT, was considered statistically significant (*p* < 0.05).

## Results

**Figure 1:**
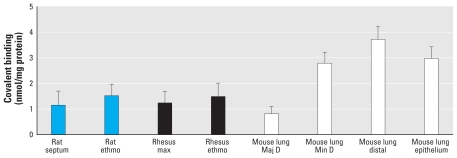
Covalent reactive metabolite binding to protein in rat, rhesus, and mouse tissue. Abbreviations: ethmo, ethmoturbinate; Maj D, major daughter; max, maxilloturbinate; Min D, minor daughter. Fresh samples of rat septum (*n* = 3), rat olfactory ethmoturbinate (*n* = 3), rhesus maxilloturbinate (*n* = 8), and rhesus ethmoturbinate (*n* = 8) were incubated with ^14^C-naphthalene (250 μM) for 2 hr at 37°C for determination of the levels of covalent reactive metabolite binding to protein. Values are mean ± SD. Reference values for tissue subcompartments of mouse lung are from Cho et al. (1994).

The specific activities of adducted proteins (nanomoles bound per milligram protein) generated in *in vitro* incubations of nasal epithelium from rats or rhesus macaques varied from 1.0 to 1.5 nmol/mg protein; we noted no statistically significant difference in the data obtained from rat olfactory and septal epithelium compared with rhesus ethmoturbinates and maxilloturbinates (Figure 1). Compared with incubations of target airway subcompartments (minor daughter and distal airway) from the mouse (Cho et al. 1994), the overall levels of adduct generated *in vitro* are slightly lower in rat and monkey nasal epithelial mucosal incubations.

Representative images from the phosphorimaging screens and complementary silver-stained gels/phosphor images indicating the spot locations chosen for MALDI dual time of flight (TOF/TOF) MS analysis are available in Supplemental Material, Figures 1 and 2 (doi:10.1289/ehp.0901333). In general, more adducts were apparent on the images from incubations of rat nasal epithelium than on those from rhesus macaques, but the patterns of major adducts were similar. Spots were matched using the alignment function of Progenesis SameSpots and Progenesis PG220 (version 2006; Nonlinear Dynamics, Durham, NC) matches were manually verified. For identification, we used only those features that could be aligned with the storage phosphor images and selected from the gel with high confidence.

We identified a large number of adducted proteins after incubation of rat nasal olfactory epithelium with naphthalene (Table 1). These included a number of redundant identifications of structural proteins (actin and tubulin), catalytic proteins (some of which are involved in energy metabolism such as ATP synthase), and a number of proteins involved in the unfolded protein response, including one of the master regulators of that response, glucose regulated protein (heat shock 70 kDa protein 5). In contrast, fewer features were identified from the gels of monkey ethmoturbinates, representing only three nonredundant proteins (Table 2). Two of these were structural proteins (actin and tubulin), and the third was a protease inhibitor with a myriad of functions.

## Discussion

The chronic bioassays conducted by the National Toxicology Program on the potential carcinogenicity of naphthalene in mice (Abdo et al. 1992) and rats (Abdo et al. 2001) have raised concerns about the possible effects of this compound on human health. In mice, an increased incidence of bronchioloalveolar adenoma was observed in females at the highest concentration tested (30 ppm) (Abdo et al. 1992), and in rats, the nasal olfactory epithelium was a prominent target after 105 weeks of exposure (Abdo et al. 2001). Long et al. (2003) observed dose-dependent increases in adenomas of the respiratory epithelium and a significant increase in olfactory epithelial neuroblastomas in male and female rats, respectively. In the olfactory epithelium, the incidence of hyperplasia and chronic inflammation was nearly 100%, even at the lowest concentration tested (10 ppm). Overall, the high degree of hyperplastic and inflammatory effects and the relative susceptibility of areas of the nasal epithelium after long-term exposures correlate well with the susceptible sites for acute toxicity (Lee et al. 2005) and raise the possibility that the tumorigenic effects of naphthalene observed in animals are related to repeated cycles of injury and repair (North et al. 2008).

The published assessments by regulatory bodies on the potential carcinogenicity of naphthalene in humans differ (e.g., International Agency for Research on Cancer 2002; National Toxicology Program 2004). Although the toxicologic data in animals are very clear, human epidemiologic studies in populations of exposed workers are uninformative. In most short-term mutagenicity assays, naphthalene is negative (reviewed by Brusick 2008). Recent evidence for the formation of DNA adducts comes both from *in vitro* studies with naphthalene metabolites (Saeed et al. 2007) and from skin-painting studies using both naphthalene and naphthalene metabolites (Saeed et al. 2009). The skin-painting studies demonstrated the presence of both depurinating and stable adducts, suggesting that this may be a possible mechanism for tumorigenesis associated with naphthalene in rodent models.

Previous studies demonstrating 10- to 100-fold differences in the rates of pulmonary microsomal formation of glutathione conjugates of naphthalene between rodents and human/nonhuman primates (Buckpitt et al. 1992; Buckpitt and Bahnson 1986) and in dissected airways of rodents and nonhuman primates (Boland et al. 2004; Buckpitt et al. 1995) suggested that humans would be considerably less susceptible to naphthalene than are rodents. Although the dramatic differences in susceptibility to naphthalene of airway epithelium in the mouse and rat appear to be related to metabolism of naphthalene by CYP2F, recent work showing that several human CYPs can metabolize naphthalene with high efficiency suggests that other CYP enzymes may catalyze the turnover of this substrate in humans (Cho et al. 2006; Fukami et al. 2008). In particular, CYP2A13, which has been reported in both lung and nasal epithelium of humans (for review, see Ding and Kaminsky 2003), shows a *K*_m_ of 36 μM and high catalytic efficiency with naphthalene. In addition, our finding of similar levels of covalent protein binding in nasal epithelium of the rodent and monkey (Figure 1), as well as observations that some (but not all) of the proteins adducted by reactive metabolites of naphthalene are the same in dissected airways and nasal epithelium of rodents and nonhuman primates (Lin et al. 2005, 2006; see also Tables 1 and 2), supports the need for additional studies on the possible sensitivity of humans to naphthalene exposure.

The concept that posttranslational modification of proteins by electrophiles begins a series of events leading to cellular necrosis was proposed by Brodie et al. (1971) some 40 years ago, and was supported by studies showing correlations between overall electrophile protein adduct formation and toxicity (for a recent review, see Hanzlik et al. 2009). The advent of new MS techniques coupled with 2D gel electrophoresis has resulted in the identification of numerous protein targets for cytotoxic agents that require metabolic activation; a current database of these targets shows many commonalities (Fang et al. 2009). Despite these efforts, a final unifying hypothesis tying the adduction of specific proteins with toxicity has not emerged, and additional work is needed to answer several questions. In those cases where adduction occurs to an abundant protein, is this protein simply acting as a sink to trap electrophiles? Does the formation of an adduct with an individual protein alter the function of that protein directly, or is the deleterious effect mediated by protein-interacting partners? These will clearly be challenging questions but, if solved, could greatly enhance our ability to more quickly and precisely assess the human risk of exposure to agents that undergo metabolic activation to protein-reactive electrophiles.

As noted above, much of the progress in identifying proteins targeted by reactive metabolites has derived from the application of 2D gel electrophoresis and more rapid MS-based identifications. Although 2D gel electrophoresis arguably provides the greatest resolving power for whole proteins of any separations technique available, there is still the potential of selecting a spot that contains two unseparated proteins, one that is low abundance and adducted and one that is much higher abundance and identified by MS. Although we cannot exclude this as a possibility in the present study, we used IPG strips with very narrow ranges to lessen the potential for misidentification. In only one case that we are aware of—with thiobenzamide—were the investigators able to show that the features removed from the gel were adducted by using isotope ratios with equal quantities of hydrogen/deuterium-labeled substrate (Ikehata et al. 2008). This was likely possible only because the adduct levels generated from thiobenzamide are considerably greater than those for other metabolically activated toxicants.

In the present study, adducts with the structural proteins actin and tubulin were isolated from both rat and monkey nasal epithelial incubations. These are high-abundance proteins in the cell that have been identified in several other studies as common targets for other reactive metabolites, including butylated hydroxytoluene (BHT) (Meier et al. 2005), benzene (Williams et al. 2002), thiobenzamide (Ikehata et al. 2008), and the model thiol-reactive electrophiles biotinyl-iodoacetamidyl-3,6-dioxaoctanediamine and 1-biotinamido-4-(4′[maleidoethylcyclohexane]-carboxyamido)butane (Dennehy et al. 2006). The formation of covalent adducts with actin and tubulin is not generally considered to be a critical step in the pathway to cellular injury because the specific activity of these adducted proteins (nanomoles bound per nanomole of protein) is likely to be quite low. However, one of the earliest signs of cellular disruption in naphthalene-treated mice involves the formation of apical membrane blebs and apparent disruption of cytoplasmic filaments (Van Winkle et al. 1999). Similar signs of disruption of cytoskeletal elements have been noted in airway epithelial cells after administration of diethyl maleate, but these changes resolved and did not lead to frank necrosis (Phimister et al. 2005). This is consistent with previous work showing that *S*-glutathionylation of cysteine-374 of actin reduces the rate of polymerization of this structural molecule (Dalle-Donne et al. 2003) and that glutathionylation of this protein leads to decreased affinity of its partner protein tropomyosin (Chen and Ogut 2006). Both naphthalene and diethyl maleate substantially deplete glutathione levels in airway epithelium, which may result in increased levels of glutathionylated actin. Reactive metabolites from naphthalene produce a double hit on actin and tropomyosin, resulting in both thiolation reactions (which are reversible) and covalent arylation reactions (which presumably are not). Ongoing work in our laboratory is evaluating the protein targets of diethyl maleate as a mechanism for distinguishing adducts that may be critical to cellular injury from those that are not.

We observed a potentially important group of protein targets in nasal olfactory epithelial incubations from the rat but not from the monkey; these proteins are responsible for protein folding and repair in the cell. Glutathione depletion is a necessary but insufficient prelude to naphthalene cytotoxicity. The loss of this key thiol leaves the cell vulnerable to intracellular reactive oxygen species that, with diethyl maleate, can be corrected by chaperones, protein disulfide isomerase, peroxiredoxins, and possibly several other proteins involved in antioxidant protection and protein folding. With naphthalene, several of these proteins, including the master regulator of the unfolded protein response, BiP/GRP78 (78 kDa glucose- regulated protein) (Townsend 2007), as well as the heat shock proteins HSP60 and HSP70, are adducted, thus potentially altering their ability to repair proteins undergoing thiol oxidation and unfolding during the early stages of naphthalene toxicity. This is consistent with findings showing marked up-regulation of the HSPs in mouse lung at early times after naphthalene treatment (Williams et al. 2003). An interesting but unproven link in possible mechanisms comes from recent work demonstrating that proteins involved in the unfolded protein response are substantially up-regulated in smokers compared with former smokers and nonsmokers (Kelsen et al. 2008).

The present study provides a list of potential protein targets and shows that some of these are similar in nasal epithelium of the rat (a known target for naphthalene toxicity) and that of the rhesus macaque. We derived several other adducted proteins from monkey nasal epithelial incubations, but these yielded unreliable identifications. We are working on methods for concentrating adducted proteins to provide greater sensitivity for these analyses. In the future, it will be important to gain additional insight regarding critical versus noncritical protein adduction. Part of this information will come from studies such as that published by Meier et al. (2007), which showed that metabolites of BHT alkylate key antioxidant enzymes and that the alkylation resulted in a decrement in enzyme function that, in turn, was consistent with the biological responses after BHT treatment.

In the present study we focused on protein adducts that are most likely related to acute cellular injury. Whether repeated cycles of injury and repair with subsequent hyperplasia are related to naphthalene tumorigenicity will need additional study. Finally, the possibility that exposure to naphthalene and other similar, volatile polycyclic aromatic hydrocarbons (PAHs) could produce lung disease other than cancer should be explored. In this regard, limited studies have reported correlations between serum levels of naphthalene and pyrene, but not other PAHs, with asthma incidence in children (Al-Daghri 2008). Moreover, there is a clear association of exposure to traffic-related pollution and asthma (Kim et al. 2005), and naphthalene is the most prevalent PAH in this mixture (Lu et al. 2005). Recent work showing that polymorphisms that result in alterations in enzymatic activities for two key enzymes involved in the metabolism of PAHs (microsomal epoxide hydrolase and glutathione transferase pi) lead to increased incidence of asthma in children living near freeways (Salam et al. 2007) provide an interesting but unsubstantiated link between PAHs in motor vehicle exhaust and asthma. The understanding of target proteins and their relationship to the pathology from naphthalene in animals should improve our ability to assess the possible risks of this compound in exposed human populations.

## Conclusion

This study shows that the amounts of covalent adduct generated from naphthalene in incubations of rhesus nasal epithelium are similar to those formed using rat olfactory and septal nasal epithelium, two sites known to be susceptible to the cytotoxicity associated with naphthalene. Many of the adducted proteins identified are the same as those observed in incubations with other target tissues. Our results are consistent with the view that nasal epithelium of the rhesus macaque may be susceptible to naphthalene cytotoxicity.

## Figures and Tables

**Table 1:** Adducted proteins identified from incubation of rat olfactory ethmoturbinate with ^14^C-naphthalene.

Gel spot	Protein name (abbreviated)	MOWSE score^a^	Peptide count^b^	Protein score CI%^c^	Accession no.^d^	Protein MW (Da)	Protein pI	Summary of function
N1	Sorcin (P30626)	133	5	100	SORCN CRILO^e^	22017.4	5.32	Calcium-binding protein
N4	Golgi autoantigen	90	24	99.951	GI: 808869^f^	372014.8	4.96	May participate in forming intercisternal cross bridges of the Golgi complex
N7	pyruvate dehydrogenase	69	7	99.596	ODPB_RAT^e^	39,336	5.94	Glycolysis
N8	mu-crystallin	135	4	100	IPI00214448	33704.1	5.34	Ammonia elimination
N9	pyrophosphatase	67	7	99.283	IPI00371957	33206.3	5.26	Signal transduction
N12	creatinine kinase	543	20	100	IPI00470288	42983.4	5.39	Energy transduction
N13	Sec 1413 protein	131	6	100	IPI00208939	46453.5	5.64	Hydrophobic ligand binding
N19	creatinine kinase	299	10	100	IPI00470288	42983.4	5.39	Energy transduction
N23	actin	259	10	100	IPI00369618	59162.6	5.67	Structural
N24	eukaryotic translation initiation factor 4A1	129	14	100	IPI00369618	46352.6	5.32	Protein synthesis initiation
N25	beta actin	447	10	100	GI: 62897625^f^	41737.8	5.37	Structural
N26	actin, cytoplasmic	420	15	100	IPI00767505	42108.9	5.31	Structural
N27	actin	753	17	100	IPI00194087	42,334	5.23	Structural
N29	actin, cytoplasmic	198	6	100	IPI00765011	59162.6	5.67	Structural
N32	actin, cytoplasmic	167	4	100	IPI00765011	59162.6	5.67	Structural
N33	actin, cytoplasmic	186	5	100	IPI00765011	59162.6	5.67	Structural
N41	ATP synthase	919	21	100	IPI00551812	56318.5	5.19	ATP catabolism
N43	ATP synthase	160	17	100	IPI00551812	56318.5	5.19	ATP catabolism
N47	heat shock protein 1A (chaperonin) (Hsp 60)	220	27	100	IPI00339148	61088.4	5.91	Stress-induced chaperone; protein folding
N51	heat shock 70 kDa 9 A (predicted)	419	9	100	IPI00363265	74,097	5.97	Protein folding; protein export; binds ATP, nucleotides, and proteins
N53	heat shock 70 kDa 9 A (predicted)	64	5	98.428	IPI00858386	74098.9	5.97	Protein folding; protein export; binds ATP, nucleotides, and proteins
N55	Vomeromodulin	87	8	99.992	IPI00209782	79527.3	5.92	Putative pheromone and odorant transporter
N56	heat shock 70 kDa, protein 5	621	15	100	IPI00206624	72473.5	5.07	Assembly of multimeric protein complexes
N57	heat shock 70 kDa, protein 5	896	27	100	IPI00206624	72473.5	5.07	Assembly of multimeric protein complexes
N61	tyrosine 3-mono-oxygenase Tryptophan 5	243	12	100	IPI00230835	28,456	4.8	Adapter protein; signal transduction
N63	tubulin	485	12	100	IPI00189795	50787.9	4.94	Structural
N64	tubulin	174	16	100	IPI00339167	50119.6	4.94	Structural
N65	tubulin	148	14	100	IPI00339167	50119.6	4.94	Structural
N66	tubulin	258	23	100	IPI00400573	49,769	4.79	Structural
N67	tubulin	464	24	100	IPI00197579	49,639	4.78	Structural
N68	protein disulfide isomerase	272	17	100	IPI00198887	56915.7	4.82	Cell redox homeostasis
N69	protein disulfide isomerase	139	15	100	IPI00198887	56915.7	4.82	Cell redox homeostasis
N70	protein disulfide isomerase	227	25	100	IPI00198887	56915.7	4.82	Cell redox homeostasis
N73	ubiquinol-cytochrome c reductase core protein 1	162	16	100	IPI00471577	57815.4	5.57	Mitochondrial electron transport
N74	ubiquinol-cytochrome c reductase core protein 1	78	19	99.939	IPI00471577	52815.4	5.57	Mitochondrial electron transport
N75	chloride intracellular channel 1	212	14	100	IPI00421995	26963.8	5.09	Macrophage activation
N76	tubulin	166	12	100	IPI0018975	5103.6	4.94	Structural
N77	tubulin	126	19	100	IPI00400573	49,769	4.79	Structural

a The probability-based MOWSE score from MASCOT.

b Peptides used for matching within the database.

c Reflects the probability that this same match would be made in a different database.

d Accession numbers are from the IPI rat database except where noted.

e From the Swiss-Prot database searching mammals.

f From the NCBI database searching mammals.

**Table 2:** Adducted proteins identified from incubations of monkey ethmoturbinate with ^14^C-naphthalene.

Gel spot	Protein name (abbreviated)	MOWSE score^a^	Peptide count^b^	Protein score CI^c^	Accession no.^d^	Protein MW (Da)	Protein pI	Function
M1	actin	333	22	100	P60709	40978.4	5.56	Structural
M2	actin	628	25	100	GI: 62897625	41737.8	5.37	Structural
M3	actin	526	23	100	P60709	40978.4	5.56	Structural
M4	tubulin	95	17	99.992	GI: 114627728	7398.9	4.72	Structural
M5	actin	107	11	100	P68133	37799.8	5.39	Structural
M6	actin	469	18	100	Q8WvW5	40477.2	5.78	Structural
M7	actin	690	23	100	GI: 4501883	41981.8	5.23	Structural
M8	actin	479	20	100	GI: 4501883	41981.8	5.23	Structural
M9	actin	181	13	100	GI: 4885049	41981.9	5.23	Structural
M10	actin	147	10	100	GI: 149758067	37799.8	5.39	Structural
M11	fibrinogen	162	12	100	GI: 109075971	36492.6	5.61	Various
M12	protease inhibitor	514	22	100	GI: 109084715	46669.9	5.55	Various
M13	actin	528	23	100	GI: 15277503	40194.1	5.55	Structural
M14	actin	588	26	100	GI: 62897671	41693.7	5.29	Structural
M15	tubulin	455	25	100	GI: 18088719	49639.9	4.75	Structural
M16	tubulin	253	20	100	GI: 23958133	49808	4.83	Structural
M17	actin	226	15	100	GI: 14250401	40978.9	5.56	Structural
M18	tubulin	338	20	100	GI: 114627728	47398.9	4.72	Structural
M19	actin	491	19	100	GI: 4885049	41991.9	5.23	Structural
M20	tubulin	314	15	100	GI: 109096498	42214.1	4.94	Structural
M21	protease inhibitor	106	11	100	GI: 109069435	42214.1	5.08	Various

a The probability-based MOWSE score from MASCOT.

b Peptides used for matching within the database.

c Reflects the probability that this same match would be made in a different database.

d From the NCBI database searching mammals.
